# Immune Thrombocytopenia Induced by Helicobacter pylori Infection: A Case Report and Literature Review

**DOI:** 10.7759/cureus.82167

**Published:** 2025-04-13

**Authors:** Almaali Alrakha, Nahla Kamal, Waleed Sherif, Ghada ElGohary

**Affiliations:** 1 Internal Medicine, Specialized Medical Center, Riyadh, SAU; 2 Adult Hematology/Stem Cell Transplant, Specialized Medical Center, Riyadh, SAU; 3 Adult Hematology/Stem Cell Transplant, Alexandria University, Alexandria, EGY; 4 Gastroenterology, Specialized Medical Center, Riyadh, SAU; 5 Internal Medicine, Ain Shams University, Cairo, EGY

**Keywords:** helicobacter pylori, h. pylori, platelet, primary immune thrombocytopenia (itp), steroid-refractory itp

## Abstract

Immune thrombocytopenic purpura (ITP) is an autoimmune disorder characterized by the production of autoantibodies targeting platelet membrane antigens, leading to platelet destruction by the reticuloendothelial system. This results in a significant drop in platelet count to 100 × 10⁹/L or lower due to the formation of autoantibodies and immune complexes. Some studies suggest a potential link between *Helicobacter pylori* infection and ITP. This report presents a case of ITP in a patient with an* H. pylori* infection. To our knowledge, this is one of the unique and interesting reported cases of such a severe platelet deficiency, where the platelet count dropped to 1,000 cells/µL in the presence of an aggressive *H. pylori* infection.

A 46-year-old male was admitted with mild gum bleeding and petechiae on his lower limbs. His medical history included chronic diabetes mellitus, dyslipidemia, and hypertension, though his clinical and vital signs were normal. Laboratory tests revealed a critically low platelet count of 3,000 cells/µL, leading to a provisional diagnosis of ITP. The patient was started on intravenous (IV) methylprednisolone (1 g for three days), IV immunoglobulin (0.4 g/kg for five days), proton pump inhibitors, calcium, and vitamin D supplements. However, there was no significant response to the treatment. Additional immunological and viral tests yielded negative results. Given this, an *H. pylori* test was conducted, which confirmed an infection. The patient was started on *H. pylori* eradication therapy. The platelet count improved to 48,000 cells/µL, but it dropped again to 1,000 cells/µL after a few days. Various treatment strategies were implemented to manage both ITP and *H. pylori*. After two months, the *H. pylori* urea breath test returned negative, and the patient’s platelet count normalized. The patient was maintained on folic acid (5 mg daily) and eltrombopag (50 mg daily), with regular hematology follow-ups ensuring stable platelet levels.

This case underscores a rare presentation of ITP associated with severe thrombocytopenia (1,000 cells/µL) and aggressive *H. pylori* infection. The findings emphasize the importance of considering *H. pylori* in the differential diagnosis of ITP and highlight the necessity of identifying underlying causes for effective treatment.

## Introduction

The autoimmune disorder known as immune thrombocytopenic purpura (ITP) is characterized by the generation of autoantibodies directed against the membrane antigens of platelets, resulting in the destruction of platelets by the reticuloendothelial system [1,2]. Adult platelets have a typical lifespan of 8-10 days, with levels ranging 150-400 × 10⁹/L in blood. The platelet count drops to 100 × 10⁹/L or below when autoantibodies and immune complexes develop in the blood [3].

Except for idiopathic ITP, secondary ITP encompasses all types of thrombocytopenia and can be inherited or acquired. Chronic infections and several autoimmune diseases are more prevalent among acquired causes of secondary ITP. Hepatitis C virus (HCV), Epstein-Barr virus (EBV), and human immunodeficiency virus (HIV) are among the chronic infections that cause immune system tolerance syndrome (ITP) [2].

In 1998, an Italian study reported that 8 out of 11 ITP patients treated with eradication treatment for *Helicobacter pylori* had a rise in platelet count [4]. This finding first highlighted the connection between *H. pylori* infection and ITP [4]. Since then, other studies have documented cases of *H. pylori* eradication [4-13]. Here, we describe an instance of ITP in a patient who had an infection with *H. pylori*. To our knowledge, this case is one of the reported unprecedented platelet deficiencies, where the platelet count reached 1,000 cells/µL.

## Case presentation

A 46-year-old male was brought to the emergency department with sudden, mild gum bleeding and petechiae on his lower limbs. He reported no symptoms of fever, weight loss, trauma, alcohol use, abdominal pain, melena, heartburn, genital ulcer syndrome, or any bleeding. His medical history included chronic diabetes mellitus, dyslipidemia, and hypertension. His medications included dapagliflozin/metformin (10/1,000 mg once daily), sitagliptin/metformin (50/850 mg twice daily), rosuvastatin (10 mg once daily), and telmisartan (40 mg once daily).

Upon examination, he was hemodynamically stable, with normal vital signs (temperature: 36.6°C, blood pressure: 118/83 mmHg, heart rate: 82 beats/minute, respiratory rate: 20 breaths/minute, and oxygen saturation of 97% in room air). He was oriented to time, place, and person. Clinical assessments of his gastrointestinal, respiratory, and cardiovascular systems were normal, and no lymphadenopathy was detected in the groin, cervical, or axial regions. Initial laboratory test results are shown in Table 1. Additionally, protein electrophoresis and peripheral blood smear showed marked thrombocytopenia. Lactate dehydrogenase, erythrocyte sedimentation rate, iron, folic acid, and vitamin B12 levels were all within normal ranges.

**Table 1:** Laboratory tests results.

Parameter	Result	Normal range
Complete blood count		
Hemoglobin	16.3 g/dL	13.5–17.5
Red blood cells	5.54 M/µL	4.5–5.9
Hematocrit	45.6%	37–53
Mean corpuscular volume	82.30 fL	80–100
Mean corpuscular hemoglobin	29.4 pg	26–34
Mean corpuscular hemoglobin concentration	35.7 g/dL	32–36
White blood cells	3.36 K/µL ↓	4.5–11
Neutrophils	29.4% ↓	35–66
Monocyte	13.4% ↑	3–10
Lymphocyte	52.4%↑	24–44
Eosinophils	3.3% ↑	0–3
Basophils	1.5%	0–2
Platelets	3 K/µL ↡	15–450
Bleeding profile		
Activated partial prothrombin time	27 seconds	26–34
Prothrombin time	12.3 seconds	12–15
International normalized ratio	0.93	0.8–1.2
Liver function tests		
Alanine aminotransferase	40 U/L	10–45
Aspartate aminotransferase	23 U/L	10–45
Albumin	4.6 g/dL	3.5–5.2
Alkaline phosphatase	58 U/L	40–129
Total bilirubin	1.2 mg/dL	0.1–1.2
Direct bilirubin	0.41 mg/dL	0.0–0.2
Gamma-glutamyltransferase	18 U/L	10–71
Kidney function tests		
Random blood sugar	134 mg/dL	70–140
Urea nitrogen	15 mg/dL	6–20
Creatinine	0.89 mg/dL	0.7–1.2
Sodium	140 mmol/L	135–145
Potassium	3.86 mmol/L	3.5–5.1
Chloride	102 mmol/L	98–107
Bicarbonate	25 mmol/L	22–29
Estimated glomerular filtration rate	103 mL/minute	>60

**Figure 1:**
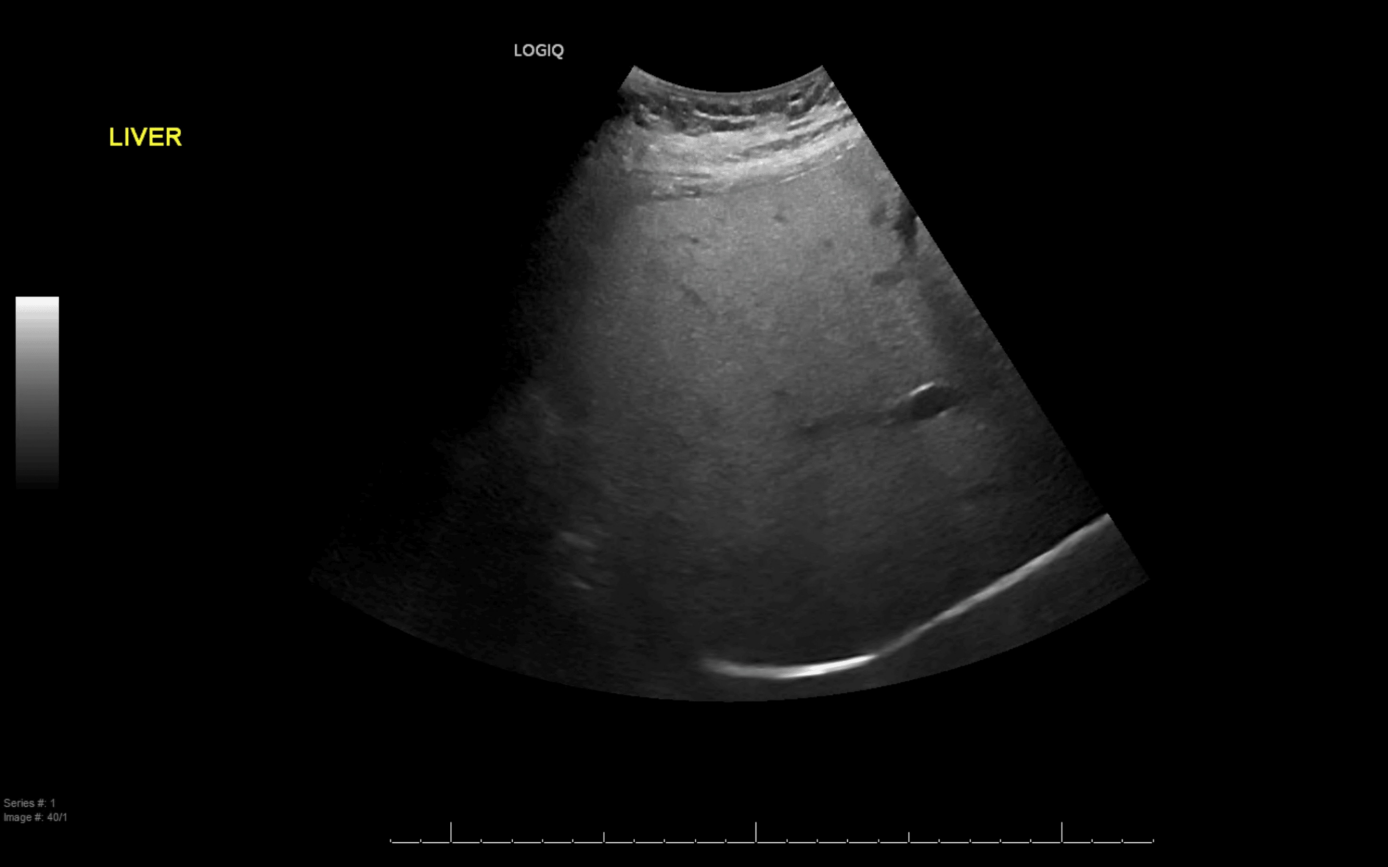
Ultrasound of the abdomen showing mild hepatomegaly with significant fatty infiltration.

**Figure 2:**
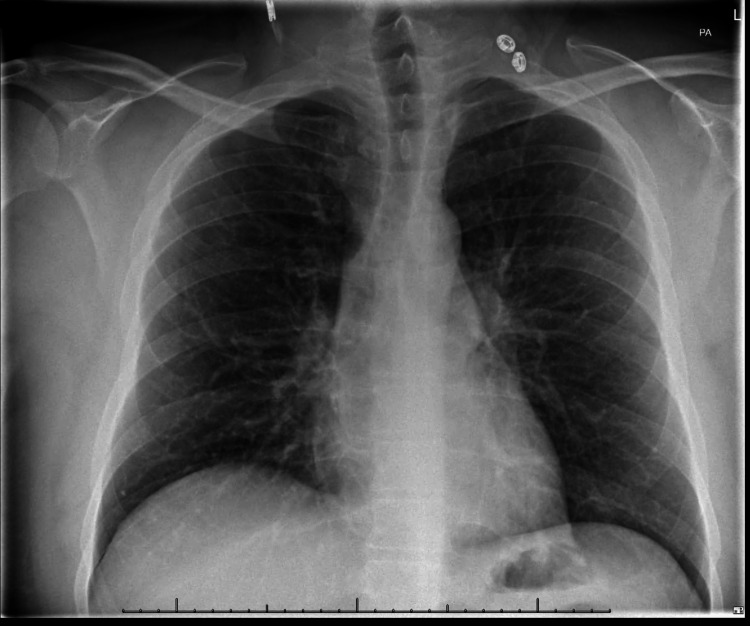
Chest X-ray.

An abdominal ultrasound (Figure 1)showed mild hepatomegaly with significant fatty infiltration, while the chest X-ray (Figure 2) appeared normal. ITP was diagnosed, and the patient was started on intravenous (IV) methylprednisolone at a dose of 1 g for three days, along with IV immunoglobulin (IVIG) at 0.4 g/kg for five days, as well as a proton pump inhibitor (PPI) and supplements for calcium and vitamin D.

Autoimmune screening tests, including lupus anticoagulant, anticardiolipin IgG and IgM, complement levels (C3 and C4), anti-nuclear antibodies (ANAs), anti-double-stranded DNA (anti-dsDNA), ADAMTS13, and rheumatoid factor (RF), all returned negative results. Serology tests for HIV, hepatitis B virus (HBV), HCV, EBV, cytomegalovirus (CMV), parvovirus B19, and quantiferon were also negative.

Additionally, he underwent an *H. pylori* stool antigen test, which confirmed an infection. Consequently, he began eradication therapy consisting of amoxicillin 500 mg twice daily, pantoprazole 40 mg twice daily, and clarithromycin 500 mg twice daily for 14 days. The stool test for occult blood was positive as well.

Platelet counts improved to 48,000 cells/µL but then fell again to 1,000 cells/µL after a few days, with other laboratory results remaining normal. The patient experienced refractory ITP that did not respond to pulse steroids or IVIG. Rituximab (375 mg/m², weekly) was introduced for four doses. He developed microscopic hematuria for a few days, which was treated with tranexamic acid. He received six units of platelet transfusion and three units of fresh frozen plasma (FFP) to improve his platelets, but the platelet count remained at 1,000 cells/µL. A thrombopoietin (TPO) agonist, eltrombopag (100 mg once daily), was added to his treatment. Bone marrow analysis showed no signs of hematological malignancy.

Despite these interventions, he continued to have refractory thrombocytopenia with a count of 2,000 cells/µL. A cycle of Velcade (bortezomib) at 2 mg subcutaneously was initiated, along with mycophenolate mofetil (MMF). Platelet levels improved to 262,000 cells/µL, but a repeated *H. pylori* urea breath test (UBT) was positive.

The patient was discharged on oral prednisolone (30 mg twice daily with a taper), MMF (500 mg twice daily), eltrombopag (50 mg once daily), levofloxacin (500 mg once daily), amoxicillin (1,000 mg twice daily), and esomeprazole (40 mg once daily) for 14 days, with a follow-up scheduled in the outpatient department.

During outpatient follow-ups, the *H. pylori* UBT remained positive twice, despite different treatment regimens. He received the third protocol for eradication, including metronidazole (500 mg three times per day), amoxicillin (1,000 mg twice daily), and PPI for 14 days. This was followed by a switch to Pylera (bismuth/metronidazole/tetracycline three capsules four times per day) for 10 days, alongside a PPI. After two months, the *H. pylori* UBT result returned negative. The patient continued taking folic acid (5 mg once daily) and eltrombopag (50 mg once daily), with regular follow-ups in hematology, and his platelet count remained within the normal range

## Discussion

Hematological conditions such as megaloblastic anaemia (resulting from a vitamin B12 shortage), iron deficiency anemia, and ITP appear to be primarily representative of the extragastric symptoms of H. pylori infection [14]. A few case studies have discussed the connection between *H. pylori* and ITP. Eight of the eleven patients who were diagnosed with ITP responded to eradication treatment, according to Gasbarrini et al. [4]. Patients with *H. pylori* infection showed platelet improvements following eradication therapy, according to Vanegas et al. [5]. On the other hand, there was no reaction to complete recovery and varied reactions to platelet resolution.

There is also worry that platelet resolution may be impacted by the degree of thrombocytopenia or the duration of the disease [6]. The ITP cases that fully recovered following *H. pylori* eradication medications are summarized in Table 2 [7-13]. We reported a case of ITP in a patient who had been diagnosed with *H. pylori* infection and had an unheard-of platelet deficit.

**Table 2:** A summary of published cases of immune thrombocytopenia caused by H. pylori infection.

Author/Year	Age/Gender	Symptoms	Platelet count	Treatment	Outcomes
Goto et al., 2001 [7]	53/Female	Idiopathic thrombocytopenic purpura	Platelet-associated IgG (PAIgG) was 695 ng/10^7^ cells	Prednisolone and splenectomy. *Helicobacter pylori* eradicated by amoxicillin, clarithromycin, and lansoprazole	PAIgG decreased to 33 ng/10^7^ cells
Tiwari et al., 2009 [8]	40/Female	Bleeding gums that had been worsening for the past 10 days, accompanied by widespread purple blotches all over the body, bleeding into the right eye, and melena	40 × 10^3^/mm^3^	Oral pantoprazole (40 mg twice daily) for six weeks. Due to *H. pylori* infection, the patient was kept on oral amoxicillin + clavulanate (625 mg twice daily), oral clarithromycin (500 mg twice daily), and oral pantoprazole (40 mg twice daily) for seven days	Following a year of treatment, the measurement was 160 × 10^3^/mm^3^
Etou et al.. 2013 [9].	41/Female	Sometimes complained of mild mucous hematochezia	6.4 × 10^4^/μL	*H. pylori* eradication therapy with amoxicillin, clarithromycin, and lansoprazole	The patient’s platelet count remained within the normal range
Hill et al., 2014 [10]	54/Female	She had no evidence of peripheral lymphadenopathy or hepatosplenomegaly	4.7 × 10^4^/μL	At first, she was given 40 mg of prednisone orally every day. Triple treatment for 14 days, consisting of 500 mg of clarithromycin taken twice a day, 1,000 mg of amoxicillin taken twice a day, and 30 mg of lansoprazole taken twice a day	Her platelet counts stayed between 15.5 and 17.6 × 10^4^/μL, which is the typical range
Marques et al., 2019 [11]	57/Male	Evolving petechial rash and gingivorrhagia within the previous month	<10 × 10^9^/L	Eradication therapy (amoxicillin 1,000 mg bid, clarithromycin 500 mg bid, metronidazole 500 mg bid, and pantoprazole 40 mg bid) was initiated immediately. It lasted for 14 days	Platelet counts were measured one month following the end of therapy, and it was 139 × 10^9^/L
Ramachandran et al., 2022 [12]	28/Male	Hematemesis	<3,000/μL	Treatment with dexamethasone and *H. pylori* eradication	251,000/µL after half a year
Sadia et al., 2022 [13]	37/Female	Bloody vomiting	4 × 10^3^/μL	During the course of 14 days, the eradication therapy included 500 mg of clarithromycin twice a day, 500 mg of metronidazole three times a day, 40 mg of pantoprazole twice a day, and 1 g of amoxicillin twice a day	Complete recovery with a 202,000/µL platelet count

Regarding the pathophysiology by which *H. pylori* could cause the development of ITP, numerous theories have been proposed. One is that antibodies against components of *H. pylori* react with antigens on the surface of platelets. Takahashi et al. reported that platelet eluates from *H. pylori*-positive ITP patients recognized the cytotoxin-associated gene A (CagA), one of the *H. pylori*-derived proteins that determines virulence [15], but another group showed that platelet eluates from *H. pylori*-positive ITP patients that reacted with glycoprotein IIb/IIIa (GPIIb/IIIa) or GPIb were unable to recognize *H. pylori* antigens [16]. The other associated mechanisms include increased production of plasmacytoid dendritic cells, which activate the host immune response due to their lamina podia and produce various interleukins, and phagocytic perturbation caused by downregulated FcγRIIB receptors and increased phagocytic activity of monocytes [17]. Additionally, platelet activation and aggregation are caused by the presence of von Willebrand factor and anti-*H. pylori* IgG on the cell membranes of different *H. pylori* strains [18]. Ultimately, the pathogenic factor CagA of *H. pylori* is neutralized by antibodies produced by the host immune system. Moreover, VacA binds to multimerin-1 on platelet surfaces to cause thrombocytopenic purpura [19]. Comparably, individuals with ITP who had a notable increase in platelet count from the pre-treatment baseline have also shown the benefit of *H. pylori* eradication therapy (triple therapy) [19].

ITP is still classified as an exclusion diagnosis owing to the lack of a specific test that can identify it. Autoantibodies against platelet glycoproteins IIb/IIIa and Ib/IX are the most often detected, which can help with diagnosis. However, it is possible that more than 50% of the patients do not have any detectable autoantibodies [20]. According to the American Society of Hematology’s (ASH) practice guidelines, individuals who present with isolated thrombocytopenia should additionally be assessed with a peripheral blood smear, HCV, HIV, and a full blood count. Individuals with abnormalities in other cell lines need to undergo further testing, including a bone marrow examination [21]. In our case, after ITP was diagnosed, we were directed to different immunological and virology tests to make a differential diagnosis for our case. These tests included the lupus anticoagulant, anticardiolipin IgG, IgM, C3, C4, ANA, anti-dsDNA, ADAMST13, and RF, all of which were negative. Serology was negative for HIV, HBV, HCV, EBV, CMV, parvovirus B19, and QuantiFERON. Finall,y we tested the patient for *H. pylori*, which led to a positive result.

It is possible to test for *H. pylori* endoscopically or non-endoscopically. Because there are non-invasive, less expensive diagnostic techniques such as UBT, stool antigen testing, and serology, some studies have recommended *H. pylori* testing for patients with persistent ITP. Testing for histology and antibiotic susceptibility can be facilitated by endoscopy combined with biopsy, which can also confirm *H. pylori* infection. Clinicians must choose which test to employ based on several factors, including clinical conditions, patient desire, pretest chance of infection, prior therapies, performance metrics, and cost [22]. No one test is thought to be the diagnostic gold standard. In our case, we depended on the *H. pylori* stool antigen test, which led to a positive result.

Life-threatening bleeding, especially cerebral hemorrhage, is the main consequence linked to ITP [23]. When the platelet count falls below 50 × 10^9^/L, as well as in patients who are having surgery or have had trauma, therapy for ITP is advised [24]. As our case had aggressive platelet deficiency, we immediately started the treatment protocol.

According to ASH, IVIG, corticosteroids, immunosuppressive medication, anti-D immunoglobulin, and splenectomy are recognized as treatments for ITP [25]. In the updated ASH (2019) recommendations, rituximab, eltrombopag (Revolade), and romiplostim have also been linked to the treatment of ITP [26]. In addition, patients with ITP are now advised to undergo the *H. pylori* eradication therapy, which entails triple therapy including PPIs (omeprazole, lansoprazole, pantoprazole) and antibiotics (amoxicillin, clarithromycin, and metronidazole) for two weeks [27]. Before the diagnosis of *H. pylori*, the patient was treated with IV methylprednisolone (1 g for three days), IVIG (0.4 g/kg for five days), a PPI, and calcium and vitamin D supplements; however, there was no response. After considering ITP secondary to *H. pylori*, we began triple therapy. His platelet count improved to 48,000 cells/µL but then dropped again to 1,000 cells/µL after a few days. He received six units of platelet transfusion and three units of FFP, and a TPO agonist, eltrombopag (100 mg once daily), was added, but, unfortunately, there was still no response.

Subsequently, he was initiated on one cycle of Velcade (bortezomib) at 2 mg subcutaneously, along with MMF, which led to an improvement in platelet levels to 262,000 cells/µL, alongside a positive *H. pylori* UBT. Upon discharge, he was prescribed oral prednisolone (30 mg twice daily with tapering), MMF (500 mg twice daily), eltrombopag (50 mg once daily), levofloxacin (500 mg once daily), amoxicillin (1,000 mg twice daily), and esomeprazole (40 mg once daily) for 14 days.

During follow-up in the outpatient department after one month, the *H. pylori* UBT remained positive twice despite different treatment regimens. He was given the third protocol of eradication, which included metronidazole (500 mg three times daily), amoxicillin (1,000 mg twice daily), and PPI for 14 days. This was followed by a switch to the fourth protocol of eradication, which included Pylera (bismuth/metronidazole/tetracycline three capsules four times daily) for 10 days, along with a PPI. Finally, after two months, the *H. pylori* UBT returned negative.

The patient continued taking folic acid (5 mg once daily) and eltrombopag (50 mg once daily), and with regular follow-ups in hematology, his platelet count remained within the normal range.

## Conclusions

This case underscores the intricate relationship between *H. pylori* infection and ITP, particularly in the unusual presentation of severely diminished platelet levels. The documented instance of a 46-year-old male patient displaying a staggering platelet count of just 1,000 cells/µL, compounded by chronic health conditions, represents an alarming manifestation of ITP that may have been exacerbated by the presence of H. pylori. The initial lack of response to standard treatments emphasizes the importance of accurately identifying underlying causes of ITP, suggesting that *H. pylori* infection may play a pivotal role in some cases. As we have seen, despite aggressive treatment protocols and supportive measures, the patient’s recovery hinged on addressing the *H. pylori* infection directly, which ultimately led to the normalization of platelet levels. This case not only enriches the existing literature on ITP but also highlights a critical need for clinicians to consider *H. pylori *infection as part of the differential diagnosis when faced with similar patients. Furthermore, it calls for a greater awareness of the potential link between gastrointestinal pathogens and autoimmune conditions, which may provide significant implications for treatment strategies. Moving forward, comprehensive diagnostic evaluations that include screening for *H. pylori* in patients with unexplained thrombocytopenia should be routinely performed, thereby promoting a more holistic approach to managing autoimmune diseases and improving patient outcomes.
